# A nomogram for predicting metabolic steatohepatitis: The combination of NAMPT, RALGDS, GADD45B, FOSL2, RTP3, and RASD1

**DOI:** 10.1515/med-2021-0286

**Published:** 2021-05-17

**Authors:** Shenling Liao, He He, Yuping Zeng, Lidan Yang, Zhi Liu, Zhenmei An, Mei Zhang

**Affiliations:** Department of Laboratory Medicine, West China Hospital, Sichuan University, Chengdu 610041, Sichuan, China; Department of Endocrinology and Metabolism, West China Hospital, Sichuan University, Chengdu, China

**Keywords:** nomogram, metabolic steatohepatitis, nonalcoholic steatohepatitis, weighted gene co-expression network analysis.

## Abstract

**Objective:**

To identify differentially expressed and clinically significant mRNAs and construct a potential prediction model for metabolic steatohepatitis (MASH).

**Method:**

We downloaded four microarray datasets, GSE89632, GSE24807, GSE63067, and GSE48452, from the Gene Expression Omnibus database. The differentially expressed genes (DEGs) analysis and weighted gene co-expression network analysis were performed to screen significant genes. Finally, we constructed a nomogram of six hub genes in predicting MASH and assessed it through receiver operating characteristic (ROC) curve, calibration plot, and decision curve analysis (DCA). In addition, qRT-PCR was used for relative quantitative detection of RNA in QSG-7011 cells to further verify the expression of the selected mRNA in fatty liver cells.

**Results:**

Based on common DEGs and brown and yellow modules, seven hub genes were identified, which were NAMPT, PHLDA1, RALGDS, GADD45B, FOSL2, RTP3, and RASD1. After logistic regression analysis, six hub genes were used to establish the nomogram, which were NAMPT, RALGDS, GADD45B, FOSL2, RTP3, and RASD1. The area under the ROC of the nomogram was 0.897. The DCA showed that when the threshold probability of MASH was 0–0.8, the prediction model was valuable to GSE48452. In QSG-7011 fatty liver model cells, the relative expression levels of NAMPT, GADD45B, FOSL2, RTP3, RASD1 and RALGDS were lower than the control group.

**Conclusion:**

We identified seven hub genes NAMPT, PHLDA1, RALGDS, GADD45B, FOSL2, RTP3, and RASD1. The nomogram showed good performance in the prediction of MASH and it had clinical utility in distinguishing MASH from simple steatosis.

## 1 Introduction

Metabolic steatohepatitis (MASH), which was once named nonalcoholic steatohepatitis (NASH), is one of the stages of metabolic-associated fatty liver disease (MAFLD), which was named nonalcoholic fatty liver disease (NAFLD). MASH is developed from simple steatosis and can progress to cirrhosis and even liver cancer. A previous study reported that the overall global prevalence of NAFLD diagnosed by imaging was approximately 25.24 and 7–30% of patients with NAFLD had NASH, indicating the overall prevalence of NASH was approximately between 1.5 and 6.45% [1]. NAFLD and NASH are becoming a global economic burden [2] and result in a poor quality of life because of complications, including type 2 diabetes [3,4], cardiovascular disease [5], and chronic kidney disease [4]. The current methods of diagnosing NASH and NAFLD are serum tests and imaging. However, these methods are not specific. Present serum biomarkers are not ideal, and all biomarkers have their limitations [6,7]. Despite NAFLD can be assessed by imaging techniques such as ultrasonography, controlled attenuation parameter, MRI-based proton density fat fraction, magnetic resonance elastography, and transient elastography, these techniques primarily evaluate steatosis and fibrosis, while inflammation is hard to differentiate [8,9]. The gold standard for diagnosing NASH is the biopsy, but the biopsy is an invasive and costly method that is not easy to be accepted by patients. Therefore, developing new, noninvasive, and reliable biomarkers is undergoing. In addition to traditional serum biomarkers, genetic biomarkers are attracting much attention. Some studies identified mRNAs or microRNAs or lncRNAs in NAFLD progression or diagnosis, for instance, UBE2V1, BNIP3L mRNAs [10], miR-192, miR-21, miR-505 [11], and lncARSR [12].

In this study, we aimed to screen potential mRNAs for the diagnosis of MASH. Differentially expressed genes (DEGs) between NASH patients and healthy controls were identified in GSE89632, GSE24807, and GSE63067. Then we constructed weighted gene co-expression modules and screened significant genes in modules mostly related to the status of NAFLD. The common genes in DEGs and significant genes in modules were considered as hub genes related to the disease. Based on the decision curve analysis (DCA) and receiver operating characteristic (ROC) curve, we validated the clinical utility of the nomogram of hub genes in predicting MASH.

## 2 Materials and methods

### 2.1 Download microarray datasets

We conducted dataset searches from the Gene Expression Omnibus (GEO) database of the National Center for Biotechnology Information (https://www.ncbi.nlm.nih.gov/geo/), up to March 1, 2020. The searches included the keywords (“NASH” OR “NAFLD” OR “nonalcoholic fatty liver disease” OR “nonalcoholic steatohepatitis” OR “non-alcoholic steatohepatitis” OR “non-alcoholic fatty liver disease”) and (organism: *Homo sapiens*).

To be included in the bioinformatics analysis, datasets had to fulfill the following criteria: (i) study type was expression profiling by array; (ii) samples were from liver tissue; (iii) studies included control and case samples. The search and selection process are shown in Figure S1. We chose datasets with the top three sample sizes for DEGs and chose datasets that included controls, steatosis and NASH samples for weighted gene co-expression network analysis (WGCNA) and validation.

The datasets GSE89632, GSE24807, GSE63067, and GSE48452 were downloaded from the GEO database. GSE63067 included two steatosis samples, nine NASH samples, and seven healthy samples [13]. GSE89632 included 20 samples with steatosis, 19 with NASH, and 24 healthy controls [14], and the clinical traits are listed in Table 2. GSE24807 included 12 NASH samples and 5 healthy controls [15]. GSE48452 included 14 samples with steatosis, 18 with NASH, 14 controls, 27 with healthy obese [16], and samples’ clinical characteristics are shown in Table S1. The clinical information of GSE63067 and GSE24807 were not available. The data that we download and analyzed were normalized by submitters. The data in each dataset was in the same batch, except GSE24807. Median-centered values in GSE24807 are indicative that the data are normalized and cross-comparable.

GSE63067, GSE24807, and GSE89632 were used to identify DEGs. GSE89632 was analyzed with the weighted gene co-expression network. Finally, GSE48452 was used to construct and validate the prediction nomogram.

### 2.2 Identify DEGs

The online analysis platform GEO2R (https://www.ncbi.nlm.nih.gov/geo/geo2r/) was used to compare two groups of samples to identify DEGs. DEGs between NASH samples and healthy controls were analyzed in the datasets GSE63067 and GSE89632 respectively. *p*-value <0.05 and log FC absolute value >1.2 were used as a filter for the datasets GSE63067 and GSE89632. Bioinformatics analysis was based on the R software 3.6. With the Combat function in the SVA version 3.5 R package, the batch effects in GSE24807 were corrected [17], and DEGs were analyzed using the limma R package. As log FC was generally large in the dataset GSE24807, *p*-value <0.05 and log FC absolute value >2 were used as a filter. The common DEGs were listed and the Venn diagram was made.

### 2.3 Weighted gene co-expression network analysis

With WGCNA R package, clusters (modules) of highly correlated genes were found and the correlation between modules external sample traits was constructed for GSE89632 [18]. First, the top 25% of the variance of probe expression was screened to WGCNA. Samples were clustered to check samples and two samples were excluded. The soft threshold power of *β* = 14 (scale-free *R* ^2^ = 0.85) was set to construct modules (Figure 2a and b). External traits were related to modules and the correlation index was calculated. Disease, one of the clinical traits, meant the status of NAFLD, including simple steatosis, NASH, and healthy. The two modules most relevant to the disease, brown and yellow modules, were chosen to identify hub genes. To explore the function of genes in brown and yellow modules, Gene ontology (GO) and Kyoto encyclopedia of genes and genomes (KEGG) analyses were performed on the Metascape database [19] (http://metascape.org/gp/index.html#/main/step1).

### 2.4 Identification of hub genes

Based on the WGCNA R package, gene significance (GS) and connectivity between genes and genes were calculated. Kwithin was the connectivity of a gene and other genes that were in the same module. GS was the correlation between gene expression and clinical data. Then, genes in the brown and yellow module whose Kwithin was top 5% and GS *p*-value for the disease was <0.05 were considered as significant genes. Hub genes were the intersection of DEGs and significant genes, which were NAMPT, PHLDA1, RALGDS, GADD45B, FOSL2, RTP3, and RASD1. To further observe the relation between hub genes and clinical data, the heatmap of hub genes and samples was drawn with the pheatmap R package.

### 2.5 Construction and evaluation of the prediction model

GSE48452 was used to construct and validate the prediction model with the rmda, rms, and pROC R package. The data of patients with NASH or simple steatosis were normalized by zero-mean normalization. The logistic regression analysis was performed, and PHLDA1 was little contributed to MASH. Therefore, we constructed a prediction nomogram for MASH which included NAMPT, RALGDS, GADD45B, FOSL2, RTP3, and RASD1, and the predicted value of the nomogram for MASH was obtained. To evaluate the nomogram, the ROC curve, DCA, and calibration plot were performed.

### 2.6 Cell culture and quantitative real-time PCR

The human normal liver cell line QSG-7701 was obtained from the Cell Bank of Type Culture Collection of the Chinese Academy of Sciences, Shanghai Institutes for Biological Sciences (Shanghai, China). It was cultured in RPMI-1640 medium (Gibco, USA) with 10% fetal bovine serum, and incubated at 37°C in a humidified 5% CO_2_ atmosphere. At about 70% confluence, the cells were treated with or without 0.2 mM free fatty acid (palmitic acid:oleic acid = 1:2; Sigma, USA). After 16 h treatment, the cells were collected for further experiments.

Total RNA was extracted from collected cells using miRNeasy Mini Kit (Qiagen, Germany) according to the manufacturer’s instructions. The reverse transcription was performed with Reverse Transcription Kit (Qiagen, Germany) and the cDNAs were quantified by real-time PCR by Roche LightCycler96 using QuantiNova SYBR Green PCR Kit (Qiagen, Germany). Primers used for qRT-PCR are listed in Table S2. qRT-PCR was carried out with the condition of 2 min for initial denaturation, 45 cycles for denaturation at 95°C for 10 s, annealing and extension at 55°C for 20 s, and melting curves analysis at default procedure. Relative mRNA levels were calculated by the 2^−ΔΔCT^ method and normalized by β-actin. All operations were repeated thrice.

### 2.7 Statistical analysis

Data were reported as mean ± SD. Student’s *t*-test was performed to compare differences between groups. *p* < 0.05 was statistically significant.


**Ethics and consent:** The ethics approval and consent to participate were not applicable.

## 3 Results

### 3.1 Identification of DEGs

**Figure 1:**
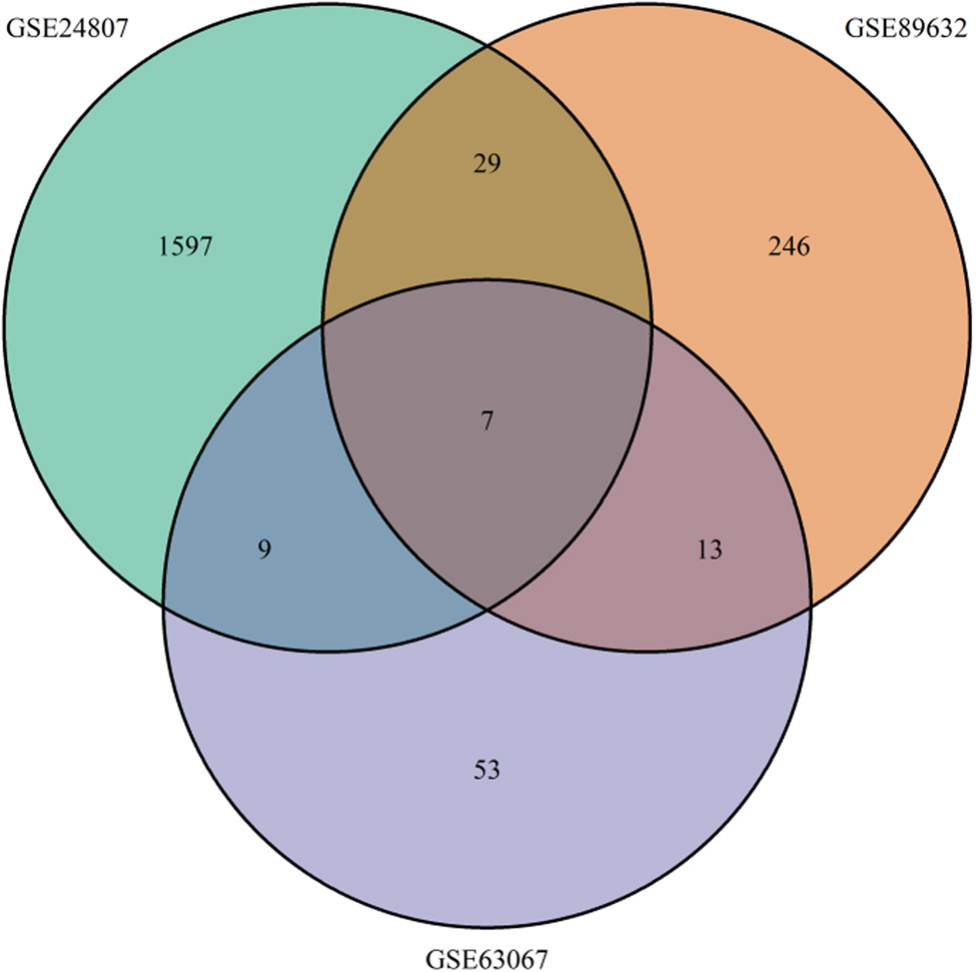
Venn diagram of differentially expressed genes (DEGs). Different colors represented different datasets, and the cross parts stood for common DEGs. Seven DEGs were shared with GSE24807, GSE63067, and GSE89632; nine DEGs were shared with GSE24807 and GSE63067; 29 DEGs were shared with GSE24807 and GSE89632; 13 DEGs were shared with GSE89632 and GSE63067.

The GEO2R and limma R package were applied to analyze DEGs. A total of 296 DEGs were screened in GSE89632 (*p*-value <0.05, log FC absolute value >1.2); 83 DEGs were screened in GSE63067 (*p*-value <0.05, log FC absolute value >1.2); and 1,643 DEGs were screened in GSE24807 (*p*-value <0.05, log FC absolute value >2). The common DEGs were presented in a Venn diagram (Figure 1) and extracted in a list (Table S3).

### 3.2 Construction of weighted gene co-expression module

**Figure 2:**
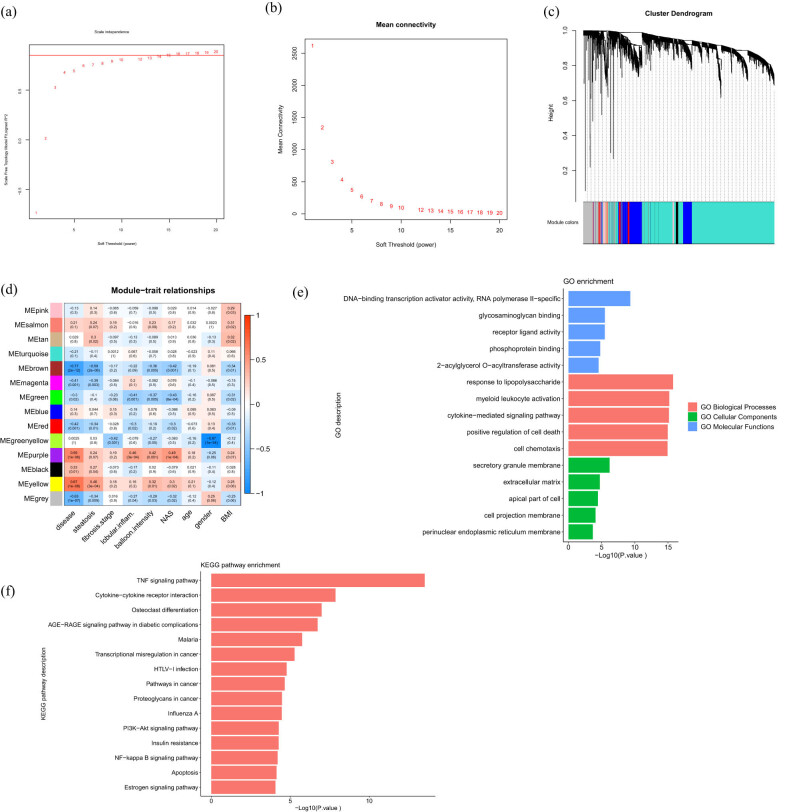
Construction of weighted gene co-expression modules and the relationship between module and trait. (a) Analysis of the soft threshold, red line = 0.85. (b) Analysis of mean connectivity. (c) Cluster dendrogram based on the dataset GSE89632. Different colors represented different co-expression gene modules. (d) Heatmap of the relationship between module and clinical trait. Each column represented clinical data, and each row represented each co-expression module. Each small grid stood for each pair of the module and trait, and indicated correlation index and *p*-value. Blue and red represented negative correlation and positive correlation, respectively. The deeper the color of the grid, the stronger the correlation. (e) Top five significant GO MFs, BPs, and CCs enriched by genes in brown and yellow modules. (f) KEGG pathway enriched by genes in brown and yellow modules (top 15).

After the WGCNA, the cluster dendrogram is as shown in Figure 2c. There were 14 modules shown in different colors. Gray module represented genes that cannot be clustered. Brown module was mostly related to disease (correlation index = −0.77, *p*-value = 2 × 10^−12^) and steatosis (correlation index = −0.59, *p*-value = 2 × 10^−6^). Yellow module was second related to disease (correlation index = 0.67, *p*-value = 1 × 10^−6^) and steatosis (correlation index = 0.46, *p*-value = 3 × 10^−4^) (Figure 2d). Brown module and yellow module had a negative and positive relation to disease, respectively. Brown module inhibited the progress of NAFLD, while the yellow module promoted the progress of NAFLD. As a result, brown and yellow modules were selected to further analyze.

There were 551 genes in the brown module and 412 genes in the yellow module. GO and KEGG pathway analyses for genes in the two modules were performed. The top five significant GO molecular functions (MFs), biological processes (BPs), and cellular components (CCs), and top 15 KEGG pathways were demonstrated (Figure 2e and f). The enriched BPs were primarily associated with response to lipopolysaccharide, leukocyte activation, cytokine, and cell death, while MF mainly enriched in DNA-binding transcription activator activity. CC chiefly enriched in secretory granule membrane and extracellular matrix. The KEGG analysis indicated that the principal enriched pathways were TNF signaling pathway, cytokine–cytokine receptor interaction, osteoclast differentiation, and AGE–RAGE signaling pathway in diabetic complications. Together, these genes highlight inflammation and inflammatory cytokines.

### 3.3 Identification of hub genes

Genes in the brown and yellow modules were calculated Kwithin and GS *p*-value. The Kwithin of repeated genes were averaged. Screened by Kwithin and GS *p*-value, brown module and yellow module owned 27 and 20 significant genes, respectively. Intersected by significant genes and DEGs, hub genes, seven in total, were NAMPT, PHLDA1, RALGDS, GADD45B, FOSL2, RTP3, and RASD1 (Table 1).

**Table 1:** List of hub genes. From top to bottom, hub genes in each module were arranged by the Kwithin from large to small

Module	Hub genes	Alias	Ensembl ID	Definition
Brown	NAMPT	PBEF, PBEF1, VF, VISFATIN	ENSG00000105835	Nicotinamide phosphoribosyltransferase
	PHLDA1	DT1P1B11, PHRIP, TDAG51	ENSG00000139289	Pleckstrin homology like domain family A member 1
	RALGDS	RGDS, RGF, RalGEF	ENSG00000160271	Ral guanine nucleotide dissociation stimulator
	GADD45B	GADD45BETA, MYD118	ENSG00000099860	Growth arrest and DNA damage inducible beta
	FOSL2	FRA2	ENSG00000075426	FOS like 2, AP-1 transcription factor subunit
Yellow	RTP3	LTM1, TMEM7, Z3CXXC3	ENSG00000163825	Receptor transporter protein 3
	RASD1	AGS1, DEXRAS1	ENSG00000108551	Ras-related dexamethasone induced 1

**Figure 3:**
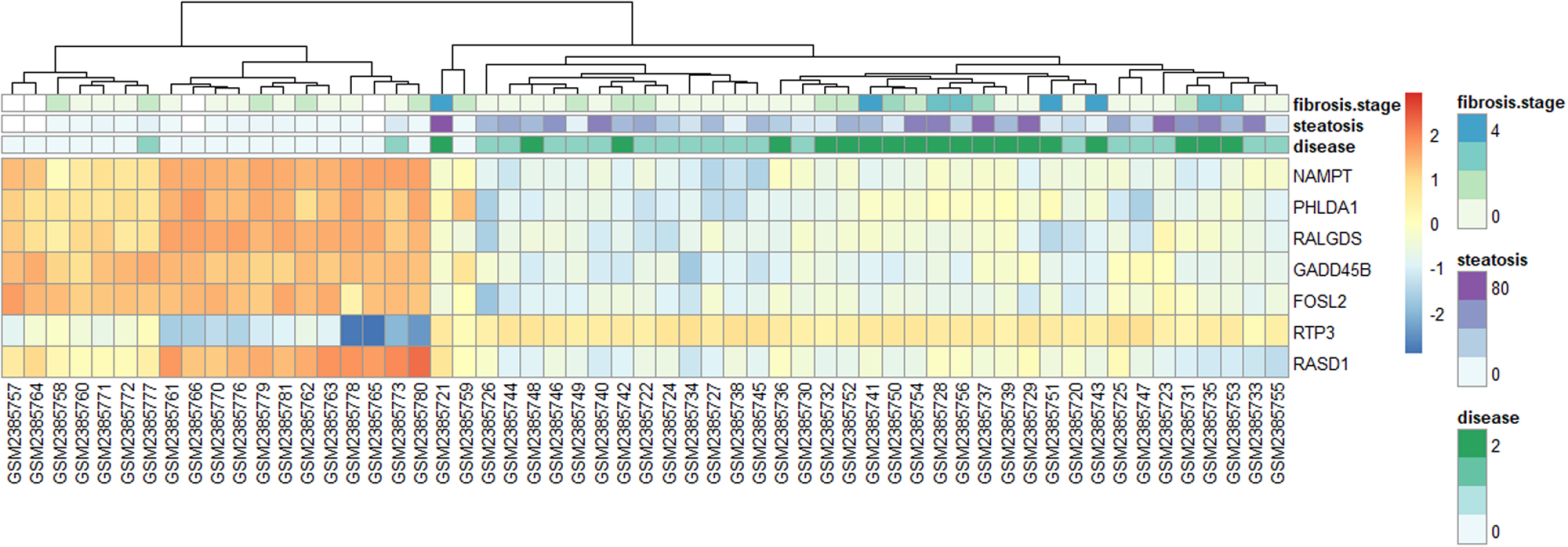
Heatmap of hub genes and samples. Each column represented one sample in the dataset GSE89632, which was annotated by clinical data in different pairs of colors. Samples were clustered. For disease, 0 (white), 1, 2 (green) represented normal sample, steatosis sample, NASH sample respectively. For steatosis, 0 (white) to 80 (purple) represented steatosis percentage. 0 (white) to 4 (blue) represented the fibrosis stage. Each row represented each hub gene. The expression of each hub gene in each sample was presented by red to blue. Red and blue represented high expression and low expression, respectively.

The heatmap of hub genes and samples is shown, which aimed to further study the relationship between hub genes and clinical data (Figure 3). NAMPT, PHLDA1, RALGDS, GADD45B, and FOSL2 were all in the brown module, with a lower expression for steatosis and NASH samples and with a higher expression for normal samples. RTP3 in the yellow module was in high expression for steatosis and NASH samples, while RASD1 in the yellow module was in low expression for steatosis and NASH samples.

### 3.4 Clinical traits and the expression of hub genes

Through the above analysis, we finally kept 19 samples with NASH, 20 samples with simple steatosis, and 18 controls in the dataset GSE89632. The clinical characteristics and the expression of hub genes are shown in Table 2. There was no difference in age and gender, and patients with NASH or simple steatosis had higher BMI than healthy controls. The steatosis of hepatocytes, fibrosis stage, lobular inflammation severity, ballooning intensity, and NAS indicated increasing histological severity from simple steatosis to NASH. The expression of hub genes was higher in samples with NASH than in healthy controls (*p*-value <0.01). The expression of NAMPT, RALGDS, GADD45B, FOSL2, RASD1, and RTP3 did not statistically differ between NASH and simple steatosis, while the expression of PHLDA1 was higher in NASH than in simple steatosis (*p*-value <0.05).

**Table 2:** Clinical data and the expression of hub genes in dataset GSE89632. Values given are mean (SD) or numbers of valid cases

Clinical traits	*n*	NASH	*n*	Simple steatosis	*n*	Healthy controls
Age (years)	19	43.47 (12.76)	20	44.70 (9.14)	18	38.67 (11.14)
Male, % (*n*)	19	47.4% (9)	20	70% (14)	18	44.4% (8)
BMI (kg/m^2^)	18	31.77 (5.45)	19	28.78 (4.23)	18	26.21 (4.00)
Steatosis (% of hepatocytes)	19	45.00 (26.45)	20	34.00 (24.37)	14	0.39 (0.74)
Fibrosis stage, 0/1/2/3/4 (*n*)	19	4/5/2/4/4	20	17/3/0/0	14	9/5/0/0
Lobular inflammation severity, 0/1/2/3 (*n*)	19	0/11/6/2	19	19/0/0/0	6	6/0/0/0
Ballooning intensity, 0/1/2 (*n*)	19	0/13/6	20	20/0/0	14	14/0/0
AST(U/L)	19	58.79 (28.11)	20	27.25 (8.51)	18	21.28 (5.94)
ALT (U/L)	19	83.47 (39.59)	19	50.84 (17.62)	18	20.94 (11.50)
Triglycerides (mmol/L)	17	2.38 (2.46)	18	1.52 (0.99)	15	0.96 (0.40)
Total cholesterol (mmol/L)	17	4.98 (1.23)	18	4.99 (1.17)	15	4.67 (1.09)
Fasting glucose (mmol/L)	17	6.18 (2.77)	17	5.71 (1.09)	18	5.03 (0.48)
HbA1c	16	6.04% (1.07%)	16	5.49% (0.44%)	18	5.41% (0.50%)
NAS, 0–8	19	4.84 (1.17)	19	1.68 (0.75)	6	0.00
NAMPT	19	13.31 (0.21)	20	13.31 (0.55)	18	14.63 (0.38)
PHLDA1	19	12.37 (0.43)	20	11.86 (0.80)	18	14.28 (0.38)
RALGDS	19	12.80 (0.32)	20	12.74 (0.68)	18	14.53 (0.55)
GADD45B	19	12.90 (0.23)	20	13.10 (0.60)	18	14.39 (0.15)
FOSL2	19	10.65 (0.27)	20	10.70 (0.84)	18	12.68 (0.46)
RTP3	19	14.30 (0.17)	20	14.17 (0.72)	18	12.36 (1.00)
RASD1	19	9.47 (0.72)	20	9.44 (1.10)	18	11.88 (1.07)

### 3.5 Model and the evaluation of nomogram

**Figure 4:**
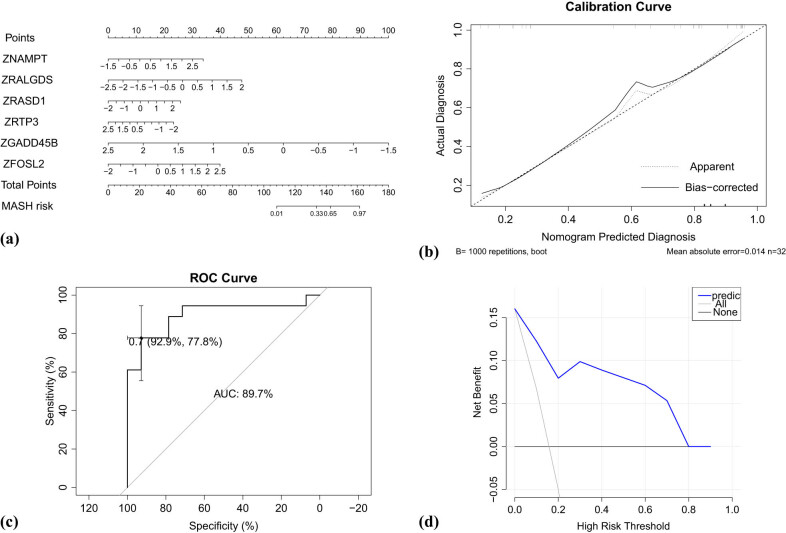
Statistical analysis for the prediction nomogram model. (a) Nomogram for distinguishing MASH and simple steatosis. All hub genes were *Z*-score normalized, and ZNAMPT meant the normalization data of NAMPT, and so on. (b) The calibration plot of the nomogram. The horizontal axis presented the predicted MASH, and the vertical axis was the actual diagnosis. The bias corrected line indicated the performance of the nomogram. (c) ROC curve of the model for the dataset GSE48452 to discriminate patients with simple steatosis from patients with NASH. (d) Decision analysis curve of the model for the dataset GSE48452. The horizontal axis was the threshold probability for NASH, and the probability of samples being NASH was calculated based on the prediction model. When the probability of the sample being NASH was more than the threshold probability, the sample was considered as NASH according to the model. The vertical axis was the net benefit. Gray line represented the net benefit of that all samples were NASH and were received the treatment for NASH. Black line represented the net benefit of that all samples were simple steatosis and forwent the treatment for NASH. Blue line represented the net benefit of that NASH samples predicted by the model received the treatment for NASH.

GSE48452 was used to construct a logistic regression model. The model of NAMPT, RALGDS, GADD45B, FOSL2, RTP3, and RASD1 is shown as the nomogram (Figure 4a). The calibration curve of the nomogram presented when the possibility of actual NASH was 0.4–0.8, and the nomogram might underestimate the probability (Figure 4b). The nomogram showed good prediction performance in differentiating steatosis and MASH (Figure 4c), and the area under the curve (AUC) was 0.897.

DCA calculated the net benefit without additional clinical information, such as life-years saved or quality of life improved [20]. In Figure 4d, where the threshold probability for MASH was 0–0.8, the prediction model was valuable, which meant the net benefit of the prediction model was better than treat all and treat none. Where threshold probability was more than 0.8, the prediction model was of no value, which meant the prediction model was as the same result as treat none. Therefore, the prediction model could be used for the dataset GSE48452 if the threshold probability was 0–0.8.

### 3.6 The relative expression of hub genes *in vitro*

**Figure 5:**
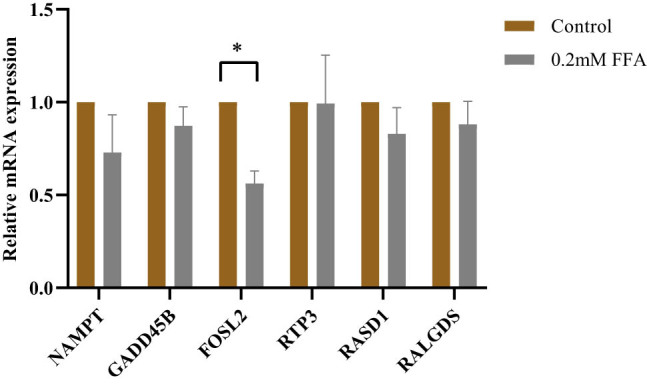
Relative expression of NAMPT, GADD45B, FOSL2, RTP3, RASD1, and RALGDS in QSG-7011 cells with or without FFA (*p < 0.05; mean ± SEM; *n* = 3).

The expression of hub genes in QSG-7011 cells with or without FFA was quantified by qRT-PCR, and the results are shown in Figure 5. The relative expressions of NAMPT, GADD45B, FOSL2, RTP3, RASD1, and RALGDS in QSG-7011 cells with 0.2 mM FFA were lower than controls, but only the expression of FOSL2 was statistically significant.

## 4 Discussion

In the study, we used the analysis of DEGs and WGCNA to identify hub genes. Not a single gene, but clusters of highly correlated genes were detected and related to clinical traits with the use of WGCNA [18]. Through GO and KEGG analyses, we found genes in brown and yellow modules enriched in inflammation such as leukocyte activation, cytokine interaction, and TNF signaling pathway. This further confirmed that the two modules are indeed related to the progression of MASH.

GEO2R analysis obtained the DEGs between NASH samples and controls in the three datasets. These datasets were from different platforms, and so we used common DEGs to reduce the effect of different platforms. We combined common DEGs and significant genes for disease status in WGCNA to get hub genes that were able to predict NASH and distinguish NASH from steatosis. Finally, seven genes overlapped, which were NAMPT, PHLDA1, RALGDS, GADD45B, FOSL2, RTP3, and RASD1. A prediction model was constructed through logistic regression analysis. Then, we visualized the model and performed the ROC curve and decision curve analyses for the model.

Samples with NASH were different from simple steatosis in histology, including steatosis of hepatocytes, lobular inflammation severity, and ballooning intensity. Although there was no significant statistical difference in the expression of hub genes, the decision curve revealed the prediction model had clinical utility, and it had net benefit within certain risk probability. The area under the ROC curve was 0.897, and the curve illustrated that the sensitivity of the model was superior to specificity. However, we did not compare other diagnostic methods for MASH with our model, and whether the model was better than other diagnostic methods still need to be reevaluated [21].

In our study, we identified seven hub genes: NAMPT, PHLDA1, RALGDS, GADD45B, FOSL2, RTP3, and RASD1. These hub genes were considered to have a contribution to the pathogenesis of MASH. Because of the small sample size, PHLDA1 showed little contribution to MASH in regression analysis; therefore, PHLDA1 was excluded and the other six hub genes were made a logistic regression analysis. At the same time, we verified the expression of hub genes in QSG-7701 cells with FFA, and the expression of NAMPT, RALGDS, GADD45B, FOSL2, and RASD1 was consistent with the results of the bioinformatics analysis. However, the relative expression of RTP3 was lower in QSG-7701 cells with FFA than in controls, which was contrary to the WGCNA. The expression of all hub genes between groups was not statistically significant, except FOSL2, possibly because of the small sample size.

NAMPT, nicotinamide phosphoribosyltransferase, or visfatin, promotes nicotinamide to convert to nicotinamide mononucleotide (NMN). NMN finally converts to nicotinamide adenine dinucleotide (NAD), which is a vital coenzyme in cellular redox reactions in all organisms and participates in many signaling pathways [22]. NAMPT plays an important role in inflammation, and it promotes inflammation progress through NAD biosynthesis. Gerner et al. found that the inhibition of NAMPT could decrease the infiltration by inflammatory monocytes, macrophages, and T cells [23]. In our nomogram, the *Z*-score normalization of NAMPT is higher, and the points are higher, which indicates that NAMPT plays an important role in MASH. However, studies indicated that the deficiency of NAD played a role in aged NAFLD [24,25], and the high expression of NAMPT promoted the biosynthesis of NAD and indirectly reduced the risk of NASH by stimulating Sirt1/SREBP1 signaling pathway probably [26]. Therefore the effect of NAMPT in MASH still needs to be explored. However, a study revealed that the expression of NAMPT was of no difference between simple steatosis and NASH [25]. NAMPT also contributed to the regulation of insulin secretion in the pancreatic β-cells [22] and diabetes mellitus [27,28].

PHLDA1, pleckstrin homology like domain family A member 1, was a phosphatidylinositol-binding protein and it could suppress AKT [29]. Zhang et al. found that a high-fat diet decreased the expression of PHLDA1 in mice study, subsequently, other genes decreasing, and indicated PHLDA1 was an early biomarker of steatosis [30]. JAK2-STAT3 pathway may induce PHLDA1 expression and these proteins probably play a significant role in TLR2-mediated immune and inflammation [31].

RALGDS, Ral guanine nucleotide dissociation stimulator, is an activator of RalA. RalA and RALGDS are important to Ras-induced oncogenic transformation of cells [32]. GADD45B, growth arrest and DNA damage inducible beta, participated in p38 and JNK MAPK pathways to positively regulate apoptosis [33]. GADD45B was abundant in the kidney, liver, and lung. GADD45B was controversial in cell stress response, and it may be protective or harmful [34,35]. FOSL2, FOS like 2, AP-1 transcription factor subunit, one of FOS proteins, was implicated as regulators of cell proliferation, differentiation, and transformation. FOSL2 played an important role in diverse disease processes, mostly through the TGF-β signaling pathway [36,37]. RTP3, receptor transporter protein 3, is specific to the liver, and its expression in other tissues is little [38]. RTP3 was probably a novel candidate gene for femoral neck bone because of the significant association with hip fracture [39]. RASD1, Ras-related dexamethasone induced 1, was an activator of G-protein signaling [40]. RASD1 was probably involved in hepatic insulin resistance [41].

The study contributed to understanding the molecular mechanism of MASH from the perspective of mRNA and provided potential biomarkers for the prediction of MASH. These potential biomarkers showed good performance in predicting MASH and had clinical utility in distinguishing MASH from simple steatosis. Because the biopsy is affected by the quality of the material taken and the experience of doctors, the results of the biopsy may not fully reflect the condition of the patient. By detecting the expression of hub genes in liver cells, a predicted value is calculated by the model and it can help doctors objectively evaluate the patient’s disease status to a certain extent according to the cut-off value, and provide a reference index for less experienced doctors. Although there is still a long way before clinical application, it provides some new targets for future work.

However, the relation between hub genes and MASH or MAFLD has been studied little. It needs further study to provide more precise clinical information about diagnosis and progression. The limitations of our study should be aware of. The samples we used were not large enough. These datasets were not suitable for joint analysis as they were from different platforms. The clinical information of GSE24807 and GSE63067 were not available, which might affect the results. The baseline data of hub genes were not available, and so no comparison with baseline gene expression was made. Our model was from liver tissue, and the specificity for MASH was good. However, the expression of the model in serum needs to be observed for further evaluation.

In conclusion, NAMPT, PHLDA1, RALGDS, GADD45B, FOSL2, RTP3, and RASD1 were identified as the hub genes in the progress of MAFLD. The combination of six genes could act as a potential diagnostic model for MASH and have clinical utility in distinguishing MASH from simple steatosis. However, clinical studies with large samples are needed to further research the applicability of the model in the diagnosis for MASH.

## Figures and Tables

**Figure A1:**
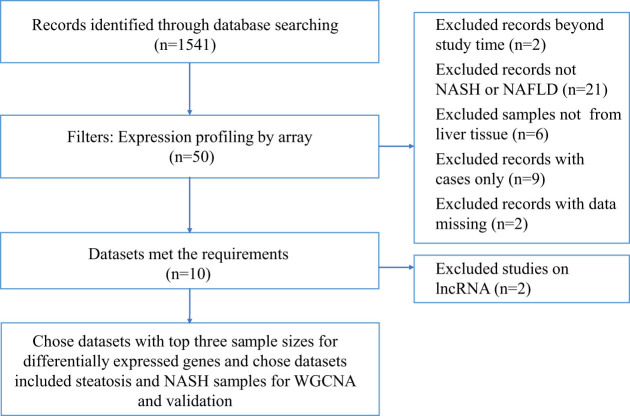
flow chart of screening datasets.

## Abbreviations

AGE–RAGE: advanced glycation end product–receptor of advanced glycation end product
BMI: body mass index
DCA: decision curve analysis curve
DEGs: differentially expressed genes
FC: fold change
FOSL2: FOS like 2, AP-1 transcription factor subunit
GADD45B: growth arrest and DNA damage inducible beta
GO: gene ontology
GS: gene significance
KEGG: kyoto encyclopedia of genes and genomes
MAFLD: metabolic-associated fatty liver disease
MASH: metabolic steatohepatitis
MRI: magnetic resonance imaging
NAD: nicotinamide adenine dinucleotide
NAFLD: nonalcoholic fatty liver disease
NAMPT: nicotinamide phosphoribosyltransferase
NAS: metabolic-associated fatty liver disease activity score
NASH: nonalcoholic steatohepatitis
NMN: nicotinamide mononucleotide
PHLDA1: pleckstrin homology like domain family A member 1
RALGDS: ral guanine nucleotide dissociation stimulator
RASD1: ras-related dexamethasone induced 1
ROC: receiver operating characteristic
RTP3: receptor transporter protein 3
SREBP1: sterol regulatory element-binding protein 1
TNF: tumor necrosis factor
WGCNA: weighted gene co-expression network analysis

## Appendix

**Table A1:** Clinical data of dataset GSE48452. Values given are mean (SD) or numbers of valid cases

Clinical traits	*n*	NASH	*n*	Simple steatosis	*n*	Healthy controls
Age (years)	18	45.48 (8.93)	14	41.60 (11.22)	13	51.80 (19.21)
Male, % (*n*)	18	22.22% (4)	14	28.6% (4)	13	30.8% (4)
BMI (kg/m^2^)	18	45.97 (12.96)	14	48.28 (6.42)	13	25.10 (3.97)
Steatosis (% of hepatocytes)	18	71.94 (16.28)	14	35.74 (22.00)	13	0.69 (1.18)
Fibrosis stage, 0/1/2/3/4 (*n*)	18	3/11/0/2/2	14	10/4/0/0	12	8/3/1/0/0
Inflammation severity, 0/1/2/3 (*n*)	18	0/9/6/3	14	12/2/0/0	13	12/1/0/0
NAS	18	5.06 (0.87)	14	1.71 (0.83)	13	0.77 (0.28)
Bariatric surgery, NA/after surgery/before surgery (*n*)	18	14/1/3	14	2/5/7	13	11/2/0

**Table A2:** RT-PCR primers for mRNA expression measurements

Gene name	Forward	Reverse
NAMPT	TTGCTGCCACCTTATC	AACCTCCACCAGAACC
GADD45B	TGACAACGACATCAACATC	GTGACCAGAGACAATGCAG
FOSL2	CCAGATGAAATGTCATGGC	CTCGGTTTGGTAGACTTGGA
RTP3	CCTTCGCCAGGTTCCAGT	GACTTCTCCTCACTCCAGTTCAT
RASD1	CGACTCGGAGCTGAGTATCC	GGTGGAAGTCCTCGATGGTA
RALGDS	TCCCAGCTGAGTCCCATCGA	TCACTAACCCCCGTCTTGCATG
β-actin	CTGGAACGGTGAAGGTGACA	CGGCCACATTGTGAACTTTG

**Table A3:** Common differentially expressed genes in the datasets

Datasets	Total	Common differentially expressed genes
GSE24807 GSE63067 GSE89632	7	MBNL2, RTP3, PHLDA1, FOSL2, NAMPT, SPSB1, CASP4
GSE24807 GSE63067	9	BBOX1, COL1A1, CHI3L1, MCL1, PLIN1, ENO3, TSLP, CCDC71L, LGALS8
GSE24807 GSE89632	29	TGM2, ATF3, ANXA13, RAB26, CALCA, CYP7A1, KLF6, ANXA9, C2orf82, IER3, ZFP36, CSF3, GRAMD4, DUSP10, GADD45B, IVNS1ABP, SLC22A7, IGFBP1, SLITRK3, RASD1, RRP12, RAB27A, BCL3, MT1A, TRIM15, CYR61, SIK1, C2CD4A, IFIT3
GSE63067 GSE89632	13	NR4A2, SERPINB9, CEBPD, IGFBP2, RALGDS, S100A8, BCL2A1, AVPR1A, IL1RN, S100A12, PEG10, CD274, BIRC3
